# Potential Association of Hypernatremia With Mortality in Patients With Acute Kidney Injury and COVID-19

**DOI:** 10.7759/cureus.27530

**Published:** 2022-07-31

**Authors:** Mahendra Atlani, Ashok Kumar, Abhijit P Pakhare, Abhishek Singhai, Ramesh Gadwala

**Affiliations:** 1 Nephrology, All India Institute of Medical Sciences, Bhopal, IND; 2 Biochemistry, All India Institute of Medical Sciences, Bhopal, IND; 3 Community and Family Medicine, All India Institute of Medical Sciences, Bhopal, IND; 4 General Medicine, All India Institute of Medical Sciences, Bhopal, IND; 5 Internal Medicine, All India Institute of Medical Sciences, Bhopal, IND

**Keywords:** aki and hypernatremia, aki outcome, covid 19 and aki, aki mortality, covid 19 and hypernatremia

## Abstract

Background

The outcome of acute kidney injury (AKI) in patients with COVID-19 and the factors associated with its outcome, including mortality, are understudied among the Indian population.

Objective

The objective of this study is to determine the outcome of AKI in a cohort of patients with COVID-19 admitted to medical wards and associated intensive care unit (ICU) and the factors associated with its outcome, including mortality.

Method

This is a retrospective study of patients with COVID-19 and AKI admitted to a tertiary care hospital. A total of 1765 patients were admitted to a hospital with COVID-19 between March 23, 2021, and June 30, 2021, during the second wave of the pandemic chiefly attributed to SARS-Co-V-2 lineage B.1.617. Patients with AKI for whom a nephrology call was sought for management (N=60) were included. Measurements carried out were the stage of AKI, co-morbidities, ICU admission, mechanical ventilation, lab parameters, and mortality. We classified AKI by comparing the highest to lowest recorded serum creatinine in hospital and staged AKI based on the Kidney Disease: Improving Global Outcomes (KDIGO) system. We further developed stepwise logistic regression models to find independent factors associated with mortality.

Results

Out of the 1765 patients hospitalized with COVID-19, a total of 60 (3.4%) patients with AKI were referred to nephrology for management. The observed mortality in this cohort was 41/60 (68.3%). AKI stage 3 was observed to be the most common (78.3%). Based on a univariate analysis of association, age, chronic kidney disease, admission to ICU, the requirement for vasopressor and ventilation, lactate dehydrogenase (LDH) DH, liver function tests (LFT), hypernatremia, and leucocytosis were associated with the mortality of patients (p<0.05) with AKI and COVID-19 infection. Multivariate analysis using logistic regression led to the identification of hypernatremia (OR 5.24 {0.95-42.31}) and multiple co-morbidities (OR 2.59 {1.03-8.75}, p<0.07) as potential factors independently associated with mortality.

Conclusion

The study indicates the potential association of hypernatremia with mortality in AKI, along with the simultaneous presence of multiple co-morbidities with COVID-19. As the statistical power of the association is weak, we are claiming the association as potential only. It needs to be confirmed in other larger studies.

## Introduction

Acute kidney injury (AKI) in patients infected with COVID-19 has been found to be a serious illness; the mortality rate among patients with AKI has been reported to vary between 20 and 76% [1]. Kidney injury results from direct effects as well as from systemic effects of COVID-19 illness such as septic shock. Nephrotoxins also contribute to kidney injury [2,3]. Several electrolyte abnormalities (dysnatremias, hypo- and hyperkalemia, and hypocalcemia) are reported in patients with COVID-19 infection and may be a manifestation of renal injury due to COVID-19 illness or may be a result of other pathophysiology, e.g., syndrome of inappropriate antidiuretic hormone (SIADH) [4,5]. The severity of AKI, age, and various scores of patient illness, such as Sequential Organ Failure Assessment (SOFA), Acute Physiology and Chronic Health Evaluation (APACHE II), D-dimers, bilirubin, oliguria in patients on renal replacement therapy (RRT), etc., have been reported to be predictors of mortality in the subgroup of patients with AKI [2,6,7]. Factors associated with mortality in patients with AKI and COVID-19 illness are understudied and under-reported in India [8, 9]. Our objective was to determine which factors conferred an increased odds of death for patients admitted to a hospital with COVID-19 and AKI. In this article, we report the single-center outcome of an Indian cohort of patients with AKI and COVID-19 infection admitted to the tertiary care center during the second wave of the pandemic.

## Materials and methods

Study design and settings

The study was conducted at a tertiary care center, All India Institute of Medical Sciences in Bhopal, India, that was converted into a "COVID-only" hospital between March 23, 2021, and June 30, 2021, during the second wave of the pandemic to provide care to COVID-19 patients only. The most dominant strain during the second wave in India reported was SARS-Co-V-2 lineage B.1.617 [10]. We planned a retrospective medical record review to find the outcome of COVID-19 patients with AKI and the factors associated with mortality. The study was approved by the internal ethics committee of the All Indian Institute of Medical Sciences, Bhopal. The consent of the subjects was waived off by the Institutional Human Ethics Committee (IHEC), considering the retrospective nature of the study and the evaluation of data on a de-identified basis.

Study participants

The inclusion criteria were: 1) age between 18 and 80 years, 2) COVID-19 diagnosed by positive RT-PCR, (3) patient should have the presence of AKI either on admission or during stay for which a nephrology consultation was sought, and (4) patient should have been admitted to wards or peripheral ICUs between March 23, 2021, and June 30, 2021.

Study variables

AKI was defined according to the Kidney Disease: Improving Global Outcomes (KDIGO) clinical practice guideline, based on changes in serum creatinine [11]. We reported the stages as per the KDIGO framework as stages 1, 2, and 3. These correspond to 1.5-1.9 times their baseline creatinine, 2-2.5 times their baseline creatinine, and 3 or more times their baseline creatinine or newly required dialysis, respectively. Urine output was not considered due to the limited collection of relevant data in routine inpatient care. Particularly, ward patients were unlikely to have corrected timed input and output calculations. Change in creatinine was calculated by comparing the peak creatinine with the lowest creatinine during hospitalization, under the assumption that the lowest creatinine would best represent baseline kidney function. In addition, we assessed AKI on admission (pre-hospitalization AKI) by comparing the admission creatinine with the lowest creatinine during hospitalization. This was done to determine whether AKI was present at the time of admission. Pre-hospitalization AKI was defined as an increase of creatinine by greater than 0.3mg/dL. We calculated this by subtracting their admission creatinine from their lowest recorded creatinine during the hospital stay.

We used Acute Disease Quality Initiative (ADQI) 16 Workgroup [12] definitions of AKI recovery to classify AKI recovery, wherein persistent AKI is characterized by the continuance of AKI by serum creatinine or urine output criteria (as defined by KDIGO) beyond 48h from AKI onset. Complete reversal of AKI by KDIGO criteria within 48h of AKI onset characterizes rapid reversal of AKI and acute kidney disease (AKD) is defined as a condition wherein criteria for AKI stage 1 or greater persists for seven or more days after an exposure.

Other exposures included demographic factors, factors of COVID-19 infection severity (need for vasopressors, ventilator, need for ICU, length of admission), co-morbidities, and lab parameters including hypernatremia defined as serum sodium >145 mEq/Lt and severe hypernatremia as >152 mEq/Lt..

Outcome

Primary outcomes included mortality within the hospital for the studied hospitalization event due to COVID-19 patients with AKI.

Statistical methods

We described patient characteristics using median (25th-75th percentile) for numerical variables and frequency (percentage) for nominal variables. We stratified the various demographic, clinical, and biochemical factors across those who died or were discharged. The distribution of these variables was compared using a chi-squared test in case of nominal variables and the Mann-Whitney test in case of numerical variables. Then, looking at the results of these comparisons, we identified the factors for performing a multivariate analysis. We constructed stepwise logistic regression models, wherein variable selection was based on biological plausible previously described associations with AKI in both COVID-19 and general medical literature as well as factors selected from the univariate analysis. These included ages, multiple co-morbidities, chronic kidney disease (CKD), AKI stage, hypernatremia, white blood cell (WBC) counts, and deranged liver functions (serum bilirubin and serum glutamic-oxaloacetic transaminase (SGOT). We reported odds ratios (ORs) with 95% confidence intervals. Further, model diagnostics was performed for assessing model fit. Lastly, all analyses were done using R version 4.0 (R Foundation, Vienna, Austria).

## Results

Basic characteristics

The study included 60 patients, after the exclusion of five patients with missing data, admitted with COVID-19 infection and AKI in this analysis, for which a nephrology consultation was sought. All patients were reverse transcription polymerase chain reaction (RT-PCR) positive. Patients with AKI who did not survive were more likely to show the following conditions: higher age, male, multiple co-morbidities, diabetes, and hypertension, admission to ICU, stage 3 AKI, the requirement of vasopressors, ventilatory support, hypernatremia, lactate dehydrogenase (LDH), WBC counts, and deranged liver functions. They also had lower absolute lymphocyte counts(ALC), renal recovery, and diastolic blood pressure. Tables 1, 2 provide more details on baseline characteristics and univariately associated factors.

**Table 1:** Baseline characteristics and univariate associations 1 - n (%); median (IQR) 2 - Fisher's exact test; Wilcoxon rank sum test; Pearson's Chi-squared test BP - blood pressure, CVD - cardiovascular disease, CKD - chronic kidney disease, COPD - chronic obstructive airway disease

Patient variable	Non-survivor's group (N=41)^1^	Survivor's group (N=19)^1^	Total	p-value ^2^
Age (years)	63.0 (57.0, 68.0)	53.0 (45.0, 64.0)		0.017
Male	31 (76%)	11 (58)	42 (70)	0.164
ICU admissions	25 (61)	4 (21)	29 (48.3)	0.004
Total stay (days)	10.0 (7.0, 15.0)	18.0 (13.5, 29.0)		0.001
Systolic BP (mmHg)	121.0 (107.0, 138.0)	120.0(116.0, 142.5)		0.418
Diastolic BP (mmHg)	65.0 (58.0, 78.0)	71.0 (67.0, 88.5)		0.072
Co-morbidity-none	11 (27)	7 (37)	18 (30)	0.418
One	13 (32)	7 (37)	20 (33.3)	
Multiple	17 (41)	5 (26)	22 (36.6)	
Diabetes	16 (39%)	6 (32%)	22(36.7)	0.578
Hypertension	15 (37%)	6 (32%)	21 (35)	0.705
CVD	7 (17%)	1 (5.3%)	8 (13.3)	0.416
CKD	2 (4.9%)	5 (26%)	7 (11.7)	0.028
COPD	4 (9.8%)	0 (0%)	4 (6.7)	0.297
Vasopressors	22 (54)	5 (26)	27 (45)	0.048
Ventilator use	33 (80)	6 (32%)	36 (60)	< .001>
Fluid balance (ml)	2700 (1350-3800)	1500 (400-2300)		0.010

**Table 2:** Lab parameters and univariate associations RT PCT - reverse transcription polymerase chain reaction, Sr. - serum, CRP - C-reactive protein, LDH - lactate dehydrogenase, SGOT - serum glutamic-oxaloacetic transaminase, SGPT - serum glutamic pyruvic transaminase, ALC - absolute lymphocyte count 1 - n (%); median (IQR) 2 - Fisher's exact test; Wilcoxon rank sum test; Pearson's Chi-squared test

Patient variable	Non-survivor's group (N=41)^1^	Survivor's group (N=19)^1^	p-value ^2^
COVID RT PCR positive	41 (68.3)	19 (31.6)	0.709
CRP (<10 mg/L)	103.0 (65.0, 147.0)	125.0 (65.0, 141.5)	0.849
LDH (105-333 IU/L)	660.0 (440.0, 800.0)	575.0 (286.5, 697.5)	0.033
Sr. potassium (3.6-5.2 mEq/Lt)	4.9 (4.2, 5.8)	4.1 (3.8, 5.2)	0.224
Sr. sodium (135-145 mEq/Lt)	147.0 (139.0, 150.0)	138.0 (135.5, 141.0)	0.007
Hyponatremia (<135 mEq/Lt)	6 (15%)	4 (21%)	0.711
Hypernatremia (>145mEq/Lt)	25 (61%)	3 (16%)	0.001
Severe Hypernatremia (>152 mEq/Lt)	8 (19.5)	2 (10.5)	0.001
Sr. bilirubin (0.1-1.2mg/dL)	1.0 (0.5, 1.5)	0.5 (0.4, 0.8)	0.044
SGOT (8-45 U/Lt)	72.0 (46.0, 201.0)	36.0 (24.0, 71.0)	0.002
SGPT (7-56 U/Lt)	73.0 (45.0, 116.0)	32.0 (22.0, 67.5)	0.002
Sr. albumin (3.4-5.4 g/dL)	2.5 (2.3, 2.9)	2.9 (2.5, 3.2)	0.079
WBC (4.5-11.0 x 10^9 ^/Lt)	20.8 (15, 26.59)	8.6 (5.42, 15.43)	<0.001
Leukopenia (<4.5 x 10^9^ /Lt)	2(4.9)	1 (5.3)	<0.001
Normopenia (4.5-11.0 x 10^9^ /Lt)	2 (4.9)	12 (63)	
Leucocytosis (>11.0 x 10^9^ /Lt)	37 (90)	6 (32)	
ALC (<1000/Per mm^3^)	390.0 (208.0, 890.0)	1,002.0 (380.0, 1,165.0)	0.062
Platelets (150-400x10^9^/L)	191 (130, 258)	150 (103, 390)	0.824
Methylprednisolone dose(mg)	120 (60,120)	0.0 (0.0, 60.0)	<0.001

Incidence of AKI

The presence of AKI was not common in our cohort of patients with COVID-19, representing only about 3.6% of the patients. Twenty-nine (48.3%) of the patients admitted to the hospital with COVID-19 eventually developed AKI, whereas 31 (51.6%) patients presented with AKI. The incidence of stage 3 AKI was the most common (n=44, 73.4%), and there was an approximately equal number of stage 1 (n=8, 13.3%) and stage 2 (n=8, 13.3%) cases; Table 3 summarizes the results.

**Table 3:** AKI details AKI - acute kidney injury

Patient variable	Non survivors (N=41)^1^	Survivors (N=19)^1^	Total	p-value ^2^
Baseline Creatinine (mg/dL)	1.1 (1.0, 1.7)	1.2 (0.9, 3.1)		0.408
AKI on admission	19 (46%)	12 (63%)	31 (51.7)	0.225
AKI stage-1	4 (9.8%)	4 (21%)	8 (13.3)	0.199
AKI stage-2	4 (9.8%)	4 (21%)	8(13.3)	
AKI stage-3	33 (80%)	11 (58%)	44 (73.4)	
Maximum creatinine (mg/dL)	3.0 (2.1, 4.3)	2.7 (1.8, 4.7)		0.573
Rapid AKI reversal	1 (1.7)	2 (3.3)	3 (5)	0.001
Persistent AKI	36 (60)	8 (13)	44 (73.3)	
Acute kidney disease	4 (6.7)	9 (15)	13 (21.7)	

Outcomes of patients with AKI

There were 41 (68.3%) deaths in our cohort, whereas the remaining 19 patients with COVID-19 and AKI survived (discharged). Twenty-five (61%) of the patients who died were admitted to ICU, while 15 (79%) of the survived patients with AKI were from wards. In the mortality group, 33 (80%) of patients had stage 3 AKI, as opposed to 11 (58%) patients in the survived group. Only 19.6% had stage 1 and 2 AKI in the mortality group; in the discharge group, 42% had stage 1 or 2 AKI. In the mortality group, 22 (54%) required vasopressors, whereas 14 (74%) in the non-mortality group were not on vasopressors. In the mortality group, 33 (80%) patients required mechanical ventilation, as opposed to 13 (68%) of the survivors. Hypernatremia was present in 25 (61%) of the patients who died, whereas it was present in only 3 (16%) of the patients who survived. Most of the patients developed persistent AKI 44/60 (73.3%), whereas AKD developed in 13 (21.7%) cases. These findings are summarized in Table 3.

Table 4 shows the results of the stepwise logistic regression analysis. At each step, variables were sequentially entered, i.e., demographic variables in Model-1, co-morbidities in Model-2, and AKI stages and biochemical parameters in Model-3 and Model-4. The initial models showed that for patients who died with AKI in hospital, the odds of death increased for those with higher age (OR 1.08 {1.02-1.15}, p=0.001), the presence of CKD on admission (OR 11.96 {1.20-188.94}, p=0.04), multiple co-morbidities (OR 2.64 {1.26-6.51}, p=0.019) but not for age, stage 2 or 3 AKI (OR 3.01 {0.40-25.67}, p=0.288) and deranged liver function tests or WBC categories of normopenia or leucocytosis (Table 4). However, on final modeling, only hypernatremia and multiple co-morbidities were associated with higher odds of death (OR 5.24 {0.95-42.31} and OR 2.59 {1.03-8.75}, respectively) but only with a trend of significance (p=0.074 and p=0.070, respectively). The value of Tjur's R2 was 0.561, which explains the model was successful in explaining 50% of the variations.

**Table 4:** Multivariate regression analysis CKD - chronic kidney disease, AKI - acute kidney injury, SGOT - serum glutamic-oxaloacetic transaminase, AIC - absolute lymphocyte count, cat - category

	Model-1		Model-2		Model-3		Model-4		Model-5	
Predictors	Odds ratio	p-value	Odds ratio	p-value	Odds ratio	p-value	Odds ratio	p-value	Odds ratio	p-value
(Intercept)	0.03	0.051	0	0.007	0	0.002	0	0.002	0	0.139
	(0.00 – 0.82)		(0.00 – 0.05)		(0.00 – 0.01)		(0.00 – 0.01)		(0.00 – 5.04)	
Age	1.08	0.011	1.06	0.064	1.06	0.117	1.06	0.099	1.04	0.375
	(1.02 – 1.15)		(1.00 – 1.14)		(0.99 – 1.15)		(0.99 – 1.15)		(0.96 – 1.14)	
Female sex	0.46	0.218	0.44	0.281						
	(0.13 – 1.60)		(0.10 – 1.99)							
CKD			11.96	0.047	22.08	0.019	12.55	0.055	6.63	0.381
			(1.20 – 188.94)		(2.11 – 415.85)		(1.18 – 251.76)		(0.12 – 507.14)	
No of co-morbidities			2.64	0.019	2.98	0.023	2.98	0.027	2.59	0.07
			(1.26 – 6.51)		(1.30 – 8.90)		(1.26 – 9.12)		(1.03 – 8.75)	
AKI stage 2					0.61	0.696				
					(0.04 – 7.20)					
AKI stage 3					3.01	0.288				
					(0.40 – 25.67)					
Bilirubin							1.74	0.476		
							(0.41 – 9.76)			
SGOT value							1.02	0.135		
							(1.00 – 1.04)			
Hypernatremia									5.24	0.074
									(0.95 – 42.31)	
Normal WBC counts									0.44	0.72
									(0.00 – 41.38)	
Leucocytosis									14.18	0.228
									Observations		60		60		60		60		60	
Tjur's R2	0.172		0.277		0.315		0.379		0.561	
AIC	71.183		66.718		66.271		60.111		51.267	
log-likelihood	-32.592		-28.359		-27.136		-24.056		-18.633	

## Discussion

The study reports the outcome of COVID-19 patients (n=60) with AKI for whom a nephrology consultation was sought; therefore, a true incidence of AKI cannot be commented upon because patients for whom a renal consult was not sent may also have AKI. However, the proportion of referred patients (3.4%) does not differ much from the incidences (3.5-7.1%) reported by other Indian studies [8,13,14]. The mortality found was high at 68.3%, as reported in other studies [1,8,13].

Association on univariate analysis with factors such as age, admission to ICU, lower blood pressure, a higher requirement of vasopressors and ventilation, deranged liver function tests (bilirubin, SGOT serum glutamic pyruvic transaminase (SGPT), and albumin), and higher LDH indicates the overall critical status of the patients as found by other researchers. Although stage 3 AKI was the most common stage of occurrence, we did not find an association with mortality; it may be related to the small cohort size of our study. Severity scores for all patients were not available, so they were not included in the analysis. 

AKI on admission was also not associated with mortality; others have also reported the same [15]. This may be related to an event that can be explained by the fact that COVID-19 patients develop symptoms such as fever, cough, anorexia, diarrhea, and fatigue several days before going to the hospital [16], although no detailed analysis of the type of AKI - pre-renal, renal, or post-renal - was done in our cohort of patients. On the other hand, new onset AKI after admission to ICU in critically ill patients has been reported to be a strong indicator of increased hazard of death even in patients with lower SOFA and APACHE II severity scores on admission [17].

Hypernatremia and multiple co-morbidities, i.e., diabetes, hypertension, and cardiovascular disease, simultaneously were the only independent factor associated with higher odds of death with a trend of significance (p=0.07) in our cohort with AKI in COVID-19-infected individuals. Other studies have reported severity of AKI, age, and various scores of patient illness, i.e., SOFA, APACHE-II, D-dimers, bilirubin, and oliguria in patients on RRT, etc., to be a predictor of mortality in the subgroup of patients with AKI [2,6,7]. An Indian study found age, stage 3 AKI, and the need for mechanical ventilation to be associated with mortality on multivariate analysis [9].

The presence of hypernatremia has been reported to vary from 3.7% to 50% across various studies [17-19]. One case series reported six out of 12 patients with COVID-19 and AKI had therapy-resistant hypernatremia, all but one patient had proteinuria, and one in each group was on RRT, indicating renal injury [19]. Another Indian study reported hypernatremia (>145 mEq/L) in 26/111 (23.4%) of patients with AKI and COVID-19 [8]. 

Hypernatremia generally results from either a deficit of total body water or an inappropriately high sodium input. In our study, a cumulative fluid balance was negatively associated with hypernatremia, which makes hypervolemic hypernatremia unlikely. The high-prevailing temperature (40-42 °C) during summers in India, when the second wave of the pandemic was in progress, with high insensible losses, may have contributed to hypernatremia in the studied cohort, but it also seems less likely because of reports from other parts of the world as well [2,19]. Unphysiological high reabsorption of sodium and chloride through proximal tubular cells mediated via increased angiotensin II activity due to the down-regulation of angiotensin-converting enzyme-2 (ACE-2) receptors by SARS-CoV-2 binding has been proposed as a mechanism [19]. Future studies focusing on hypernatremia evaluation, including measurement of free water clearance, urinary electrolytes, infused electrolyte load, and urine osmolality, may guide the exact pathophysiology of hypernatremia in these patients.

Hypernatremia has been associated with adverse outcomes. When sodium plasma concentrations exceeded 150 mmol/L, the associated mortality rate was as high as 48% [20]. Our study also supports these findings. The median serum sodium (mEq/Lt) (IQR) in our study was 147.0 (139.0, 150.0) in non-survivors. Severe hypernatremia (152 mEq/Lt) was also present in significantly higher numbers in non-survivors as compared to survivors (Table 2).

In a study, median (IQR) sodium (mmol/Lt) was 144.2mmmol/Lt (139.4-146.9) in non-survivors of AKI with COVID-19 infection as compared to 137.8 (135.2-140.0) in survivors. (p=0.001). In addition, serum sodium >145 mmol/Lt was also present in a significantly higher number; 25 (41.7%) non-survivors as compared to 3 (14.3%) survivors (p=0.03) [2].

In another Indian study of patients with AKI and COVID-19, hypernatremia was more common in non-survivors (20/58 {34.5%}) as compared to survivors (06/53 {11.3%}) (p=0.016). However, the study only reported univariate associates; multivariate analysis was not carried out [8]. 

Previous studies have found an independent effect of ICU-acquired hypernatremia on mortality after adjusting for other risk factors [21,22]. Hypernatremia and the following hyperosmolar state have a variety of well-known adverse effects on physiologic functions. Therefore, the association between hypernatremia and increased mortality could be causal [20].

Multiple co-morbidities have been associated with the severity of COVID-19 infection. A meta-analysis of 120 studies and 125,446 patients, pooled estimation of all studies, revealed that 28% of COVID-19 cases had multiple co-morbidities (95% CI: 18-40%). It also reported that COVID-19 severity remained similar in patients with at least one co-morbidity (41%) or multiple co-morbidities (44%), substantially higher than in COVID-19 cases without any co-morbidity (19%). However, no association between multiple co-morbidities and mortality was reported [23]. On the other hand, another recent meta-analysis did not report an association between multiple co-morbidities and AKI [24].

Although our cohort size is small, it highlights the potential association of hypernatremia and multiple co-morbidities with mortality in AKI with COVID-19-infected individuals.

Limitations

This chart review is limited by the small sample size due to the inclusion of patients from a single center following stringent inclusion criteria. Although we have performed a multivariate analysis, we have not claimed the causality of any associations found.

## Conclusions

This small retrospective study highlights the potential association of hypernatremia and multiple co-morbidities with the mortality in AKI patients with COVID-19 infection. As the association found is weak, we are claiming the association as potential only. It should be confirmed in other larger studies. As the mortality associated with COVID-19 and AKI is very high, identification of even a single remediable association, i.e., hypernatremia, is very useful and provides an opportunity for better outcomes by management. The presence of multiple co-morbidities may also warn a poor outcome in AKI patients with COVID-19 and may guide proper resource allocation in resource-poor areas.
